# Ras homolog family member J (RHOJ): a key regulator of chemoresistance associated with epithelial-mesenchymal transition

**DOI:** 10.1038/s41392-023-01597-z

**Published:** 2023-10-02

**Authors:** Sijia Liu, Jiang Ren, Long Zhang

**Affiliations:** 1 International Biomed-X Research Center, Second Affiliated Hospital of Zhejiang University School of Medicine, Zhejiang University, Hangzhou, China; 2 Key Laboratory of Precision Diagnosis and Treatment for Hepatobiliary and Pancreatic Tumor of Zhejiang Province, Hangzhou, China; 3 The Eight Affiliated Hospital of Sun Yat-Sen University, Shenzhen, China; 4 MOE Laboratory of Biosystems Homeostasis & Protection and Innovation Center for Cell Signaling Network, Life Sciences Institute, Zhejiang University, Hangzhou, China; 5 Cancer Center, Zhejiang University, Hangzhou, China

**Keywords:** Skin cancer, Cancer therapy

**Fig. 1:**
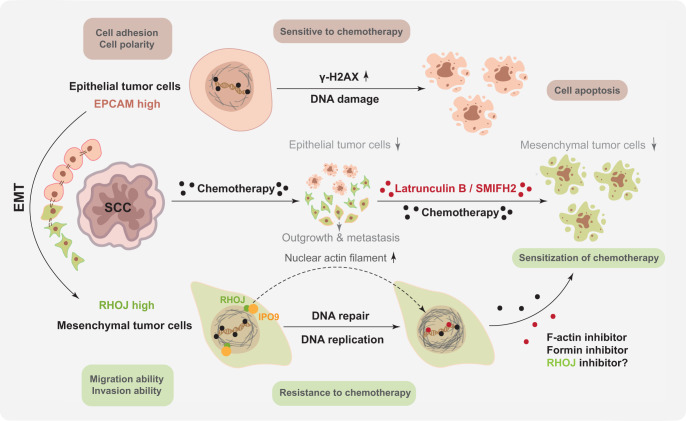
The schematic diagram of RHOJ controls EMT-associated resistance to chemotherapy. EMT is a developmental process that confers intrinsic resistance to chemotherapy in skin SCCs. Chemotherapy can induce DNA damage with increasing level of γ-H2AX in epithelial tumor cells, which will trigger cell apoptosis and inhibit tumor growth at the beginning of chemotherapy. However, mesenchymal tumor cells exhibited drug resistance and facilitated tumor outgrowth and metastasis in late stage. RHOJ was found to be highly expressed in mesenchymal tumor cells and promotes chemotherapy resistance by facilitating DNA repair and replication. IPO9 has been identified to interact with RHOJ and regulate nuclear actin filament. Inhibition of actin polymerization by F-actin inhibitor Latrunculin B or formin inhibitor SMIFH2 can sensitize mesenchymal tumor cells to chemotherapy in a RHOJ-dependent manner. RHOJ presents a promising target for novel drug development to overcome chemotherapy resistance in skin SCCs

In a recently published study in *Nature*, a group led by Cédric Blanpain identified Ras homolog family member J (RHOJ) as a key regulator of epithelial-mesenchymal transition (EMT)-associated chemoresistance in skin squamous cell carcinoma (SCC).^1^ Mechanistically, this study demonstrated that RHOJ promotes DNA repair and replication of EMT tumor cells to assist them in rapidly repairing DNA lesions induced by chemotherapy (Fig. 1).

Chemoresistance of tumor cell is a persistent problem during cancer treatment and responsible for the death of majority patients with cancer. EMT is a developmental process associated with chemoresistance in many types of cancer, in which epithelial tumor cells lose their adhesion ability and acquire a highly migratory and invasive mesenchymal phenotype.^2^ Several molecular mechanisms have been reported to responsible for cancer therapeutic resistance in cell lines, which were related with EMT in vitro.^3^ However, whether these mechanisms are account for EMT-related chemoresistance in primary tumors in vivo is still unclear.

To investigate the in vivo mechanisms underlying EMT-associated chemoresistance in skin SCCs, Debaugnies and colleagues used a transgenic mouse model (*Lgr5*^*creER*^*Kras*^*G12D*^*p53*^*cKO*^*Rosa-YFP*), which combines the expression of oncogenic *Kras* with *Trp53* deletion and *YFP* reporter expression in hair follicle lineages.^1^ This model can present spontaneous EMT during primary skin SCCs formation, enabling researchers to distinguish different cell populations response to chemotherapy. By utilizing epithelial cell adhesion molecule (EPCAM) staining to identify tumor cells undergoing EMT, it was observed that the tumors resistant to chemotherapy were highly enriched with EPCAM^−^ cells. Similar to what was found in vivo, EPCAM^−^ tumor cells were profoundly resistant to a broad range of chemotherapeutic agents in vitro. Based on previous transcriptomic and epigenomic studies of skin SCCs performed by the Blanpain group, Debaugnies et al. analyzed the potential regulators of chemoresistance in EPCAM^+^ and EPCAM^−^ tumor cells and found that RHOJ was highly expressed in EPCAM^−^ tumor cells. Using in vitro genetic gain- or loss-of-function studies, they found that overexpression of RHOJ lead to increased tumor cell survival following chemotherapy, whereas knockdown of RHOJ sensitized tumor cells to chemotherapy. Additionally, it was observed that knockdown of RHOJ resulted in decreased tumor cell proliferation and migration, while having no impact on the expression of EMT markers. To further investigate the in vivo function of RHOJ in skin SCCs, Debaugnies and colleagues generated *RHOJ* conditional knockout (KO) mice *(Lgr5*^*creER*^*Kras*^*G12D*^*p53*^*cKO*^*Rhoj*^*cKO*^*Rosa-YFP)*. After chemotherapy, RHOJ-KO mice showed more EMT tumor cell apoptosis than wild-type (WT) mice. Subsequently, they transplanted WT or RHOJ-KO EPCAM^−^ tumor cells into immunodeficient mice and provided mice with cisplatin/5FU therapy for three weeks to assess the long-term reaction of EMT tumors to chemotherapy. Results showed that WT tumors continued to grow, but RHOJ-KO tumors stopped growing during the long-term treatment. Overall, researchers concluded the major function of RHOJ in promoting chemoresistance of EMT tumor cells based on the short-term and long-term chemotherapy studies in vivo.

To elucidate the molecular mechanisms of RHOJ in regulating chemotherapy resistance in EMT tumor cells, Debaugnies et al. performed RNA sequencing and proteomic profiling of EMT tumor cells with and without RHOJ deletion.^1^ Transcriptomic and proteomic analyses revealed that RHOJ regulates genes and proteins associated with DNA repair and replication. Thereafter, they performed a series of experiments to investigate major modulators changes in the DNA damage response (DDR) pathway after chemotherapy and found that RHOJ did not modulate the activation of DDR kinases. However, phosphorylation of histone 2A.X at Ser 139 (γ-H2AX) was significantly increased after chemotherapy in RHOJ-KO EMT tumor cells compared with WT EMT tumor cells, suggesting that RHOJ prevents DNA damage accumulation in EMT tumor cells. Furthermore, bromodeoxyuridine (BrdU) incorporation analysis has been used to detect cell division history after chemotherapy and proved that RHOJ facilitates EMT tumor cells to progress through the cell cycle under chemotherapy. In addition, a higher percentage of origin firing was observed in RHOJ expressed tumor cells after chemotherapy, demonstrating that RHOJ promoted DNA replication by activating new origins of replication in EMT tumor cells during chemotherapy.

Next, RHOJ affinity purification (AP) and mass spectrometry (MS) characterization have been performed by Debaugnies and colleagues to uncover how RHOJ regulates DNA repair and replication after chemotherapy.^1^ Based on the AP-MS and co-immunoprecipitation results, they identified IPO9-a protein that mediates the nuclear import of actin-was enriched and interacted with RHOJ in EMT tumor cells after chemotherapy. This finding indicated that RHOJ may modulate the chemotherapy response of EMT tumor cells through regulating the function of nuclear actin. Using transfection of nuclear actin chromobody in different tumor cells, researchers found an increase in nuclear actin filament in EPCAM^−^ cells compared to that in EPCAM^+^ and RHOJ-KO EPCAM^−^cells, suggesting that RHOJ modulates nuclear actin polymerization. Among multiple inhibitors of actin remodeling, the F-actin inhibitor latrunculin B and the formin inhibitor SMIFH2 sensitized EMT tumor cells to chemotherapy and enhanced cell apoptosis in a ROHJ-dependent manner. All these data demonstrated that RHOJ promotes chemotherapy resistance in EMT tumor cells by activating DNA-damage response and enhancing DNA replication via the regulation of nuclear actin polymerization.

In summary, this study identified the key function and molecular mechanisms of RHOJ in the contribution of EMT-associated chemoresistance in skin SCCs, which is important foundation for the development of new strategies to overcome chemoresistance in the future. Researchers used elegant in vitro and in vivo mouse models to demonstrate that RHOJ is highly expressed in EMT tumor cells and modulates resistance to different chemotherapies, but further studies are still needed to validate their findings in clinical samples from patients with skin SCCs. Moreover, researchers found that RHOJ can also regulate tumor invasion in breast cancer,^1^ melanoma^4^ and glioblastoma,^5^ providing a direction for a new line of clinical translational research on targeting RHOJ for inhibiting tumor invasion in cancer therapy. However, the development of an effective and safe RHOJ inhibitor remains a challengeable task, and further in-depth studies are needed to discovery strategies to specifically block tumor progression with minimal side effects in healthy tissue.
